# MRONJ in breast cancer patients under bone modifying agents for cancer treatment-induced bone loss (CTIBL): a multi-hospital-based case series

**DOI:** 10.1186/s12903-023-02732-6

**Published:** 2023-02-04

**Authors:** Rodolfo Mauceri, Martina Coppini, Massimo Attanasio, Alberto Bedogni, Giordana Bettini, Vittorio Fusco, Amerigo Giudice, Filippo Graziani, Antonia Marcianò, Marco Nisi, Gaetano Isola, Rosalia Maria Leonardi, Giacomo Oteri, Corrado Toro, Giuseppina Campisi

**Affiliations:** 1 Present Address: Department of Surgical, Oncological and Oral Sciences, University of Palermo, Via L. Giuffrè 5, 90127 Palermo, PA Italy; 2 Department of Economics, Business and Statistics, University of Palermo, 90128 Palermo, Italy; 3 Regional Center for Prevention, Diagnosis, and Treatment of Medication and Radiation-Related Bone Diseases of the Head and Neck, University of Padova, 35128 Padova, Italy; 4 Oncology Unit, Azienda Ospedaliera Di Alessandria SS. Antonio e Biagio e Cesare Arrigo, 15121 Alessandria, Italy; 5 School of Dentistry, Department of Health Sciences, Unit of Oral and Maxillofacial Surgery, “Magna Grecia” University of Catanzaro, 88100 Catanzaro, Italy; 6 Department of Surgical, Medical and Molecular Pathology and Critical Care Medicine, University of Pisa, 56126 Pisa, Italy; 7 Department of Biomedical and Dental Sciences and Morphofunctional Imaging, University of Messina, 98124 Messina, Italy; 8 Department of General Surgery and Medical Surgery Specialties, School of Dentistry, University of Catania, 95123 Catania, Italy; 9 Maxillofacial Surgery Unit, Clinica del Mediterraneo di Ragusa, 97100 Ragusa, Italy; 10 University Hospital Policlinico “Paolo Giaccone” of Palermo, 90127, Palermo, Italy

**Keywords:** Osteonecrosis of the jaw, ONJ, MRONJ, Breast cancer, Cancer treatment-induced bone loss, CTIBL, Bone modifying agents

## Abstract

**Background:**

Cancer treatment-induced bone loss (CTIBL) is the most common adverse event experienced by patients affected by breast cancer (BC) patients, without bone metastases. Bone modifying agents (BMAs) therapy is prescribed for the prevention of CTIBL, but it exposes patients to the risk of MRONJ.

**Methods:**

This multicentre hospital-based retrospective study included consecutive non-metastatic BC patients affected by MRONJ related to exposure to low-dose BMAs for CTIBL prevention. Patients’ data were retrospectively collected from the clinical charts of seven recruiting Italian centres.

**Results:**

MRONJ lesions were found in fifteen females (mean age 67.5 years), mainly in the mandible (73.3%). The mean duration of BMAs therapy at MRONJ presentation was 34.9 months. The more frequent BMAs was denosumab (53.3%). Ten patients (66.7%) showed the following local risk factors associated to MRONJ development: periodontal disease (PD) in three cases (20%) and the remaining six (40%) have undergone PD-related tooth extractions. One patient presented an implant presence-triggered MRONJ (6.7%). In five patients (33.3%) no local risk factors were observed.

**Conclusions:**

This is the first case series that investigated BC patients under BMAs for CTIBL prevention suffering from MRONJ. These patients seem to have similar probabilities of developing MRONJ as osteo-metabolic ones. Breast cancer patients under BMAs for CTIBL prevention need a regular prevention program for MRONJ, since they may develop bone metastases and be treated with higher doses of BMAs, potentially leading to a high-risk of MRONJ.

## Background

Medication-related osteonecrosis of Jaw (MRONJ) is defined as an “an adverse drug reaction described as the progressive destruction and death of bone that affects the mandible and maxilla of patients exposed to the treatment with medications known to increase the risk of disease, in the absence of a previous radiation treatment” [1].

MRONJ is considered a potentially serious complication of bone modifying agents (BMAs), such as bisphosphonates (BPs) and denosumab (DNB) [2, 3]. Furthermore, several MRONJ cases have been described in cancer patients following treatment with other biological medications that have no antiresorptive activity on bone tissue (e.g. tyrosine kinase inhibitors), with or without concurrent BMAs [4, 5].

MRONJ is usually reported in two major groups of patients: (a) cancer patients with bone metastases (BM) or with multiple myeloma, usually receiving high doses of BMAs, and (b) osteo-metabolic patients under low doses of BMAs [2, 6–8]. The risk of MRONJ is considerably higher in the malignancy group, ranging between 1% and more than 20%, than in the osteo-metabolic group, less than 1% [9–12].

However, among patients at risk of MRONJ, recent data included another emerging group of patients affected by breast cancer or prostate cancer, without BM and under hormonal therapy, with peculiar clinical features. They are receiving low-doses of BMAs, at the same dosage of the osteo-metabolic patients, to prevent or to treat Cancer Treatment-Induced Bone Loss (CTIBL) [13, 14]. These patients should be considered assumable to those with osteoporosis for what concerns their MRONJ risk.

In particular, breast cancer (BC) is the most prevalent cancer worldwide [15, 16]. There were 2,3 million women diagnosed with BC and 685,000 deaths globally in 2020. BC has a prevalence estimated in 2020 (time period 5 years) of 7,8 million women [14, 15]. About 70–80% of early BC patients receive adjuvant endocrine therapy (ET). In the majority of cases, ET includes the use of aromatase inhibitors, as an upfront or switch strategy, that has well-known effect on bone demineralization [17, 18].

In patients affected by BC, since gonadotropin-releasing hormone analogues or chemotherapy and/or aromatase inhibitors reduce estrogen levels inducing early menopause, there is the risk of developing CTIBL, regardless of the risk of developing BM in advanced stages of BC [17, 18].

CTIBL is considered the most common long-term adverse event experienced by patients affected by BC receiving adjuvant ET. The BMAs are the leading therapy for the prevention and treatment of CTIBL, which is administered at the same low-dosage of osteoporotic patients, rather than that used in bone metastatic cancer and multiple myeloma patients [17, 19, 20].

In randomized controlled trials (RCT) on BC patients treated with BMAs for CTIBL prevention (mostly under zoledronic acid 4 mg IV every 6 months, or denosumab 60 mg s.c. every 6 months), the prevalence of MRONJ onset was 0–0.5%, although, at present, data are scarce and debatable [10, 21, 22]. The results of the comparisons between different molecules and drug regimens, showed an incidence of MRONJ ranging from 0.3% (in patients under daily oral clodronate) to 5.4% (under denosumab 120 mg s.c. every 4–12 weeks) [23–25].

The aim of this study was to describe the features of a series of BC females without bone metastases and affected by MRONJ under low doses of BMAs for CTIBL prevention.

## Methods

### Study design

This multicentre hospital-based retrospective study included consecutive non-metastatic BC patients affected by MRONJ related to the exposure to low-dose BMAs for CTIBL prevention. Patients’ data were retrospectively collected from the clinical charts of seven recruiting Italian centres (2 in the north and 5 in the south of Italy) between January 2016 and July 2022. In detail, these are the centres involved: Unit of Oral Medicine, “Paolo Giaccone” Policlinico University Hospital of Palermo (Italy); Unit of Maxillofacial Surgery, University Hospital of Padua (Italy); Unit of Oral and Maxillofacial Surgery, “Magna Græcia” University of Catanzaro (Italy); Unit of Dentistry and Oral Surgery, University Hospital of Pisa (Italy); Unit of Oral Pathology, School of Dentistry, University of Catania (Italy); Unit of Oral Surgery, School of Dentistry, University of Messina (Italy); Maxillofacial Surgery Unit, Clinica del Mediterraneo di Ragusa (Italy).

The study was conducted according to the ethical guidelines of the Declaration of Helsinki (1964) and its later amendments or comparable ethical standards, and it was approved by the central institutional review board (Coordinator Centre approval ID: #4/2012—“Paolo Giaccone” Policlinico University Hospital in Palermo, Italy). The study was conducted following the STROBE Statement for Observational Cohort Studies [26].

### Eligibility criteria

All BC patients scheduled to receive BMAs therapy or already exposed to BMAs for CTIBL with a suspicious or confirmed diagnosis of MRONJ, were consecutively enrolled at each participating centres from January 2016 to July 2022.

The inclusion criteria in the study cohort were the following:

- age ≥ 18 years;
- women affected by BC and treated with adjuvant endocrine therapy;
- previous or current treatment with low-dose BMAs for CTIBL prevention;
- MRONJ diagnosis according to SICMF-SIPMO clinical-radiological staging system (assessment of MRONJ radiological signs by means of computed tomography (CT) or cone beam CT (CBCT)) [6].

The exclusion criteria were:

- history of high-dose BMAs for bone metastases;
- concurrent use of anti-angiogenic agents or other drugs at risk of MRONJ onset;
- exposure to radiant therapy of the head-neck;
- suspicious or confirmed diagnosis of primary or secondary jaw cancer.

MRONJ case adjudication at the seven participating centres was performed by oral health and oral surgery specialists. Each report was supported by a qualitative case-by-case assessment procedure, which was conducted by an officially recognized drug expert, with the delivery of an adverse drug reaction report.

### Study variables

The following data were recorded in all recruited cases: demographic data; drug-related (i.e. type, dose and formulation of BMAs); clinical variables associated with MRONJ-risk (e.g. smoking habits, comorbidities such as diabetes, concomitant corticosteroids treatment); oral trigger associated to MRONJ development; site of MRONJ; disease stage according to AAOMS clinical classification and according to SICMF-SIPMO clinical-radiological staging system [6, 12].

In all cases, MRONJ diagnosis was confirmed at presentation through the clinical inspection at of the main/minor oral signs and symptoms and the detection of the radiological signs (loss of medullary bone, increased bone density, bone sclerosis) at the CT/CBTC according to the SICMF-SIPMO classification system [3, 27]. Bone biopsies were not performed, since there was no doubt regarding the suspicion of malignancies in enrolled patients [3].

The data were entered into a standardized electronic case report form in all recruiting centre.

### Statistical analysis

Continuous variables were reported as mean value and standard deviations, median, whereas categorical variables by the frequency distributions.

## Results

A total of 15 women affected by BC under BMAs for CTIBL prevention were included.

The baseline features of the cases were reported in Table 1. The mean age was 67.5 ± 11 years (median 68 years). In eight patients (8/15, 53.3%), concomitant comorbidities were the following: hypertension (7/15, 46.7%), arthrosis (1/15, 6.7%), and diabetes mellitus (2/15, 13.3%). Five patients were under corticosteroid therapy (5/15, 33.3%). Only one patient was a smoker (1/15, 6.7%). In the remaining seven patients (7/15, 46.7%) no comorbidities or co-medication were recorded.

**Table 1:** Study population features

Pt ID	Age	MRONJ- related drugs	Duration (months)	Cumulative dose (mg)	Systemic disease	Local risk factors for MRONJ	MRONJ site	MRONJ stage (AAOMS)	MRONJ stage (SICMF - SIPMO)
#1	62	Denosumab*	30	300	–	–	Maxilla	2	1
#2	88	Denosumab*	36	360	Hypertension	Dental extraction	Mandible	1	1
#3	59	Clodronate	24	19,800	Hypertension	Dental extraction	Mandible	1	1
#4	69	Alendronate	6	1680	Arthrosis	Periodontal disease	Mandible	2	1
#5	83	Denosumab*	12	120	Hypertension, diabetes	–	Mandible	1	2
#6	52	Alendronate	84	23,520	–	Dental extraction	Mandible	2	2
#7	55	Denosumab*	24	240	–	Periodontal disease	Maxilla	1	2
#8	52	Denosumab*	24	240	–	–	Mandible	1	2
#9	67	Oral ibandronate	10	1500	–	Dental extraction	Mandible	2	2
#10	58	Denosumab*	84	840	–	Peri-implantitis	Mandible	3	3
#11	68	Clodronate	84	16,800	–	–	Mandible	3	3
#12	72	Alendronate	18	5040	Hypertension	Dental extraction	Maxilla	3	3
#13	69	Alendronate	52	14,560	Hypertension	–	Maxilla	3	3
#14	81	Denosumab*	24	240	Hypertension, diabetes	Dental extraction	Mandible	2	2
#15	78	Denosumab*	24	240	Hypertension	Periodontal disease	Mandible	1	2

*Denosumab: 60 mg s.c. every 6 months

Regarding the adjuvant ET, most of the patients (9/15, 60%) received aromatase inhibitors (anastrozole, letrozole, or exemestane); two patients (2/15, 13.3%) received selective estrogen receptor modulator (tamoxifen). The remaining four patients (4/15, 26.7%) received a sequence of more agents or unspecified drugs.

Regarding BMA type, 8 patients received DNB (53.3%) (60 mg s.c. every 6 months), 4 patients used alendronate (26.7%) (70 mg os every week), 2 patients used clodronate (13.3%) (600 mg i.m., every month), and 1 patient used ibandronate (6.7%) (150 mg os monthly). The mean duration of BMA therapy at MRONJ presentation was 35.7 months (± 26.3 months, median 24 months). No patient was treated with BMAs prior to breast cancer diagnosis nor with BMAs for metastasis.

Ten patients (10/15, 66.7%) showed the following local risk factors for MRONJ development: periodontal disease (PD) in three cases (3/15, 20%), six (6/15, 40%) have undergone PD-related tooth extractions, and one patient presented an implant presence-triggered MRONJ (1/15, 6.7%). In five patients (5/15, 33.3%) no local risk factors were observed (Table 1).

**Fig. 1:**
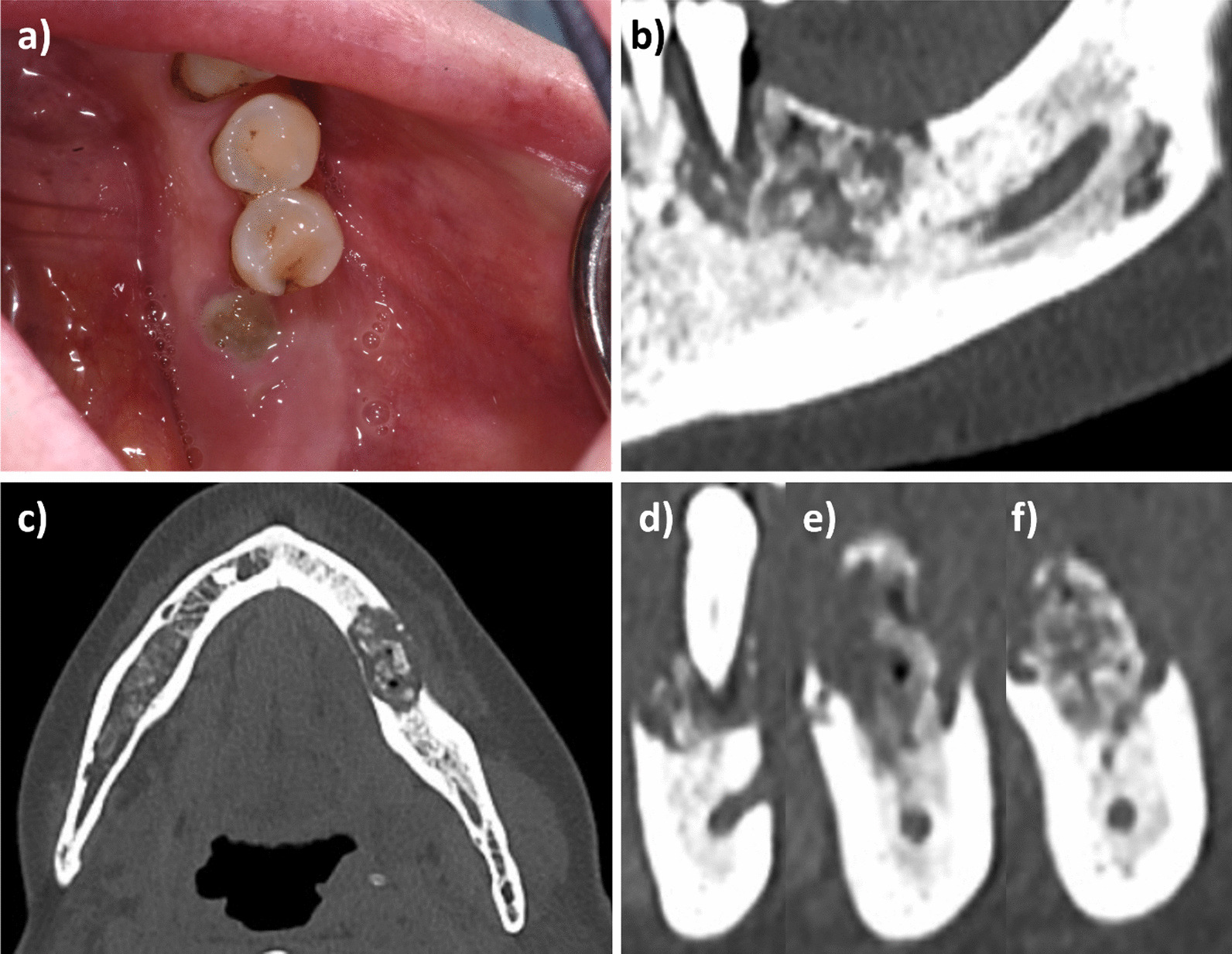
MRONJ stage 1 SICMF-SIPMO, lower jaw: **a** clinical view; **b**–**f** CBCT scan sections

The mandible was the most frequent affected site (11/15, 73.3% versus 4/15, 26.7% of MRONJ of the upper jaw) (Fig. 1). According to the AAOMS staging system [12], MRONJ cases were classified as it follows: 6 patients were in stage 1 (40%), 5 patients in stage 2 (33.3%), and 4 patients in stage 3 (26.7%). While, according to SICMF-SIPMO staging system [6], the stage distribution of MRONJ was: 4 cases of stage 1 (26.7%), 7 in stage 2 (46.7%), and 4 in stage 3 (26.7%)(Table 2).

**Table 2:** Details of patients affected by breast cancer under bone modifying agents for CTIBL

	N. 15 BC patients with CTIBL (%)
Median age	68
Mean age ± SD	67.5 ± 11
Comorbidities	Yes 8 (53.3)
	No 7 (46.7)
Diabetes	2 (13.3)
Hypertension	7 (46.7)
HCV-related hepatopathy	0
Arthrosis	1 (6.7)
Cardiovascular diseases	0
Corticosteroid therapy	5(33.3)
*Site of MRONJ*	
Mandible	11 (73.3)
Maxilla	4 (26.7)
Mandible and Maxilla	0
Oral trigger for MRONJ	Yes 10 (66.7)
	No 5 (33.3)
Tooth extraction	6 (40)
Periodontal disease	3 (20)
Peri-implantitis	1 (6.66)
Prosthetic trauma	0
*MRONJ stage according to AAOMS*	
1	5 (33.3)
2	6 (40)
3	4 (26.7)
*MRONJ stage according to SICMF–SIPMO*	
1	5 (33.3)
2	6 (40)
3	4 (26.7)

The most frequent symptoms and signs at presentation were pain (10/15, 66.7%), followed by bone exposure (9/15, 60%) and soft tissue inflammation and suppuration (9/15, 60% and 8/15, 53.3%, respectively).

## Discussion

Medication-related osteonecrosis of the jaw (MRONJ) occurs more frequently in patients receiving high doses of bisphosphonates or denosumab (RANKL-inhibitor) for metastatic cancer.

At present, a growing number of observations on MRONJ cases are emerging in patients exposed to low-dose BMAs for osteoporosis [28].

Low-dose BMA have been recently recommended for the prevention and treatment of cancer treatment-induced bone loss (CTIBL) in patients with solid tumor (breast or prostate cancer) without bone metastases.

In this scenario, these patients are considered an emerging category at risk of MRONJ development, sharing simultaneously common features of both cancer patients and osteo-metabolic patients.

The present study investigates for the first time the features at presentation and risk factors associated to MRONJ development in a series of breast cancer (BC) patients exposed to BMAs therapy for CTIBL prevention.

Cancer treatment-induced bone loss (CTIBL) has been found to be the most common long-term adverse event experienced by patients affected by BC with a peak of incidence in postmenopausal age [13].

Fifteen cases of MRONJ in patients affected by BC, without BM, under a low dose of BMAs for CTIBL prevention were collected and described.

Adjuvant ET represents the standard of care in hormone receptor-positive BC patients, which represent about 75–80% of all BC diagnoses, as it was shown to significantly reduce the risk of recurrence and cancer-related death. Adjuvant ET consists of two main drug regimens: estrogen receptor modulators and aromatase inhibitors [13]. Women affected by BC treated with ET as mainly adjuvant therapy or in premature iatrogenic menopause represent a particular subtype of patients at high-risk of fracture, due to the onset of CTIBL. As described in different guidelines [17, 29], BPs and DNB are the two classes of BMAs used in clinical practice with similar efficacy in preventing CTIBL, and they are routinely prescribed with the same dosage used in osteo-metabolic patients (low-dose BMAs) [13].

In recents RCT on BC patients without BM under a low dose of BMAs for CTIBL prevention, the incidence of MRONJ was observed between 0% and 0.5 [10, 21, 22].

As well as BC, also prostate cancer places a high burden on patients and healthcare systems, recently affecting over 1.4 million men worldwide each year [14]. Most of advanced prostate cancer patients receive Androgen Deprivation Therapy (ADT). ADT causes a rapid disruption of bone remodelling balance, which leads to net bone loss. Bone loss, as well as disruption of bone microarchitecture, continues throughout the duration of ADT and ongoing CTIBL in men with prostate cancer is superimposed upon normal age-related bone loss [30]. Hormone-sensitive advanced prostate cancer patients (without or with bone metastases) should receive low-dose BMAs to prevent CTIBL, whereas patients with castration-resistant disease and bone metastases should receive high-dose zoledronic acid or denosumab, together with anticancer treatments [17]. An attempt to adopt high-doses of denosumab (120 mg every 4 weeks) in prostate cancer patients without metastases failed to reach approval, also due to a high rate (5%) of MRONJ [17, 31].

BC patients and prostate cancer patients, both under low doses of BMAs for CTIBL prevention, are hence an emerging category at risk of MRONJ, who share simultaneously some characteristics of both cancer patients and osteo-metabolic patients. Furthermore, intermediate doses of BMAs have been administered in patients enrolled in RCTs evaluating both the prognostic effect of BMAs as adjuvant treatment in non-metastatic breast cancer, and the risk-reduction of CTIBL development as secondary endpoint.

If considered data from literature, we can consider only findings from osteo-metabolic patients under low doses of BMAs and from the same geographical area: 87 cases of MRONJ were described (mean age of 70.7 ± 9.8 years, median 72); 42 patients (42/87, 48.3%) had various general health comorbidities (e.g. diabetes mellitus, hypertension); and in 33 cases (37.9%) oral triggers for MRONJ development were detected [7]. Regarding the BPs, 77/87 (88.5%) patients were treated with alendronate (a weekly dose of 70 mg orally); the mean duration of BMAs therapy at MRONJ presentation was 44.9 (± 35.5 months, median 38 months). Bone exposure was the main presenting sign (82.8%). MRONJ was classified according to AAOMS [12], as it follows: 15 patients in stage 0 (17.2%), 12 in stage 1 (13.8%), 53 in stage 2 (60.9%) and 7 in stage 3 (8.1%). Tooth extraction for dental/periodontal diseases, was the most common trigger factor for MRONJ (57/87 patients; 65.5%) [7].

In another Italian study, fifty-three female patients affected by osteoporosis were enrolled in the study (mean age of 71.9 ± 10.2 years). Twenty-two subjects (41.5%) had hypertension, seven (13.2%) had diabetes and five (9.4%) had rheumatoid arthritis. Eight subjects were smokers (15.1%). The majority of the sample (45 subjects, 84.9%) was treated with alendronic acid (70 mg/week). Most of the lesions were located in the mandible (74%); bone exposure was found in 40 subjects (75.4%). MRONJ was classified according to AAOMS [12], as it follows: 7 in stage 1 (13.2%), 39 in stage 2 (73.6%) and 7 in stage 3 (13.2%). Tooth extraction for dental/periodontal diseases, was the most common trigger factor for MRONJ 29/53 patients (54.7%) [32].

In a third Italian study, 36 patients receiving BPs for the treatment of osteoporosis were described. There were 32 females (88.8%), and the mean age of the patients was 72.8 (± 10.1). Cardiovascular disease was the most detected comorbidity (23/36; 63.9%), followed by anxiety and/or depression, diabetes and lipid disorders. The most commonly administered BPs was alendronate (n = 26; 72.2%). The most common location of MRONJ was the mandible (29/36; 80.6%). MRONJ was classified according to AAOMS [12], as it follows: 4 in stage 0 (11.1%); 18 in stage 1 (50%), 11 in stage 2 (30.6%) and 3 in stage 3 (8.3%). The presence of a potential oral trigger was recorded in 24 (66.7%), with the most common being dental extraction [33].

The patients under BMA therapy for CTIBL could show some assumed systemic risk factors for MRONJ similar to those of cancer patients with BM, such as chemotherapy or steroids intake, whereas at the same time the prevention of CTIBL is based on a low dose of BMAs, with the same therapeutic scheme of osteo-metabolic patients [2, 6, 8].

In this case series, regarding the local risk factors, PD and PD-related tooth extraction were both reported (15.38% and 38.46% of patients enrolled, respectively). On the basis of current literature, these two variables could be considered as a “*unicum*” trigger of the most frequent dental disease is PD, implying the disease and its extreme resolution [2, 8, 34]. In detail, tooth extraction has been described as one of the major risk factors for the onset of MRONJ; however, it is indicated only in the presence of endodontic and/or periodontal diseases, in patients at risk of MRONJ, when a conservative approach is not possible, and a good tooth prognosis is not guaranteed. Hence, dental infections are basically the main reason for dental extraction, and they are local risk factors already present for long periods before surgical procedures (e.g. severe periodontitis will be a determinant for a tooth with poor prognosis). Indeed, among local risk factors, the role of periodontal infections and the oral microbiome is becoming increasingly outstanding in MRONJ onset. These conditions are associated with inflammatory responses, that may directly or indirectly affect the alveolar bone and stimulate bone resorption, and so, under the effect of BMAs, the MRONJ onset [34–37]. Furthermore, there is increasing evidence that signs and symptoms of dental/periodontal infection are significantly associated with histological alveolar bone necrosis even prior to dental extractions and MRONJ development [34, 38].

Based on the literature and the present findings, it is our opinion that it is possible to distinguish at least three main common categories of patients at risk of MRONJ [3, 8, 13, 39, 40]:

- *cancer patients with BM or myeloma patients*; generally receiving BMAs at high doses and more frequently (e.g. every four weeks), often associated with anticancer agents (chemotherapy, endocrine therapy, immunotherapy, antiangiogenics, and other biological agents). This is a group at high-risk of MRONJ.
- *cancer patients (e.g. BC patients without bone metastases, and hormone-sensitive prostate cancer patients, with or without bone metastases) at risk of non-metastatic bone fractures due to CTIBL*; generally receiving BPs or DNB to reduce the CTIBL, and/or to improve prognosis (“adjuvant” treatment of prostate and breast cancer patients). In the absence of robust data, this population (if with the same dosage of BMAs) is considered assumable to that one with osteoporosis for what concerns their MRONJ risk and must not be confused with the category a);
- *patients suffering from osteoporosis and other non-malignant diseases*, receiving BMAs low regimens. This is the group usually at the lowest MRONJ risk, unless the BMA treatment is prolonged.

All patients at risk of MRONJ should be subjected to primary preventive measures (even after commencing BMAs), with the aim to maintain and/or re-establish as soon as possible a sufficient level of oral health [3, 41, 42]. The preventive measures in cancer patients with BM or multiple myeloma should be done strictly before the administration of MRONJ-related medications, and in osteo-metabolic patients within its first six months. Furthermore, the patients taking BMAs should undergo periodic dental visits for early diagnosis of MRONJ: every four months for patients assuming a high dose of BMAs with/without antiangiogenics medication intake; every six months for patients assuming a low dose of BMAs [3, 9].

To date, data on MRONJ onset in BC patients and hormone-sensitive prostate cancer patients under low doses of BMAs for CTIBL prevention are still scarce and uncertain. Moreover, no recommendations for MRONJ have been published dedicated to BC patients under low doses of BMAs for CTIBL prevention or treatment. Apparently, they could be assimilated, with regard to MRONJ prevention workflows, to osteo-metabolic patients.

Another point of attention is that for BC females it should be taken into account also that they could previously have received BMAs at low doses before CTIBL prevention, to treat or prevent primary osteoporosis, increasing their BPs cumulative dose or BMAs time of assumption.

Noteworthy, it is important to remember that BC patients may develop bone metastases along their clinical history [43]. The risk of MRONJ onset in these patients will most likely increase drastically over time due to the development of BM that requires BMAs at high doses. Consequently, BC patients already treated with low doses BMAs (due to CTIBL) could be later treated with high doses of BMAs in combination or not with anti-angiogenic medication after developing BM. Furthermore, the previous prolonged BMA treatment could lead to a high-risk of MRONJ in a potentially short time after the start of high dose BMA therapy.

From a practical point of view, if a given cancer patient under CTIBL develops BM, before the assumption of high dose BMAs, the oral condition must be re-evaluated by dental examination and, when necessary, also thanks to a new radiological dental exam. Additionally, cancer patients that develop BM will be included in a follow-up program every 4 months, instead of every 6 months. It would be helpful to perform together with dental examination professional oral hygiene [3, 41, 42].

A drawback of this study might be the retrospective set-up of the study design; however, all the centres involved have done their best to collect long-term data (over 6 years) from heterogeneous groups of patients at risk of MRONJ. Furthermore, other limitations of the present study are the small sample size, and the lack of data on endodontic diseases.

## Conclusion

In our opinion, BC patients under low doses of BMAs for CTIBL prevention should be considered assumable to those with osteoporosis for what concerns their MRONJ risk; however, there is a lack of reliable data on the incidence and prevalence of MRONJ in the study group.

In conclusion, BC patients receiving therapy for CTIBL is an emerging category of MRONJ risk, still poorly known by many clinicians, especially dentists. This scarce knowledge may lead to the possibility of overestimating the risk of MRONJ onset in these patients if included in the same cohorts of cancer patients with BM or multiple myeloma, and of putting in place excessive or overly stringent MRONJ preventive measures. On the other hand, there is the risk that other clinicians underestimate the need to upgrade the preventive protocol for MRONJ when high doses BMAs for bone metastases must be commenced.

Based on our findings, BC patients under BMAs for CTIBL prevention need a regular prevention program for MRONJ since they may later develop bone metastases and be switched from low to high dose BMAs, potentially leading in a short time to a high-risk of MRONJ.

## Data Availability

The datasets used and/or analysed during the current study are available from the corresponding author on reasonable request.
